# Supplementary material for: Protocol of a mixed (Retrospective and Prospective) longitudinal observational study of amiodarone for chronic Chagas cardiomyopathy (AMIO-CHAGAS study): Investigating immunomodulatory and trypanocidal effects
Source: PLoS One. 2026 Apr 28;21(4):e0347305. doi: 10.1371/journal.pone.0347305 (PMC13123931; doi:10.1371/journal.pone.0347305)
Supplement: Attachment — Submitted filename: Review report_ AMIO Chagas_230226.pdf [file pone.0347305.s002.pdf]

Scientific Review Report

The manuscript presents an ambispective longitudinal protocol (retrospective and prospective), entitled AMIO-CHAGAS, which evaluates the use of amiodarone in patients with chronic Chagas cardiomyopathy treated at a referral center in Brazil. Considering that Chagas disease remains endemic in this country and that Chagas cardiomyopathy represents a significant cause of disability and cardiovascular morbidity and mortality, the topic is clinically relevant.

The study population consists of patients with confirmed diagnosis of Chagas disease. The primary objective of the protocol is to evaluate the potential immunomodulatory and trypanocidal effects of amiodarone, exploring whether, in addition to its recognized antiarrhythmic effect, the drug could have an etiological impact on Trypanosoma cruzi infection.

1. Originality and Relevance

The protocol addresses a pharmacological aspect that has not yet been fully elucidated in humans: the potential immunomodulatory and trypanocidal effects of amiodarone beyond its conventional antiarrhythmic indication. Given that the available clinical evidence regarding this effect remains limited, the study aims to contribute to a relevant gap in current knowledge.

Furthermore, considering the persistent prevalence of Chagas disease and its impact in terms of cardiovascular morbidity and mortality, the exploration of additional effects of drugs already used in this population holds scientific and clinical interest.

2. Discussion and Clinical Applicability

Although the protocol proposes an innovative hypothesis regarding the potential etiological effect of amiodarone in humans, it would be advisable for the authors to more thoroughly contextualize the clinical relevance of these findings. In particular, it is important to discuss the drug's safety profile, characterized by known adverse effects, in comparison with currently established etiological therapies for Chagas disease.

It is suggested that the clinical scope of the study be more clearly defined, emphasizing the exploratory nature of the research.

3. Methodology

The methodological design is consistent with the stated objective; however, the proposed sample size (n = 90) may be limited for detecting clinically significant differences, particularly given the exploratory nature of the immunomodulatory and trypanocidal effects being evaluated.

Although the authors report having based their estimate on two previous studies and increased the calculated sample size by 20% to account for potential losses, the parameters used for a formal sample size calculation are not described. The absence of this information

makes it difficult to determine whether the study will have sufficient statistical power to support robust conclusions.

A more detailed description of the sample size calculation procedure would strengthen the methodological rigor of the protocol.

4. Ethical Aspects

The manuscript states that the study was approved by an institutional ethics committee, including the corresponding approval number. It also specifies that all participants provided informed consent prior to inclusion, and the authors declare the absence of conflicts of interest.

Overall, the protocol appears to comply with fundamental ethical standards for research involving human participants.

5. Reporting and Data Availability

The manuscript does not include an explicit statement regarding data availability. It is recommended that a specific section be incorporated clarifying whether the data will be publicly available, accessible upon reasonable request, or subject to ethical restrictions.

6. Writing and Clarity

The manuscript is clearly written and allows adequate understanding of the objective and methodological structure of the protocol. The organization is coherent and facilitates comprehension of the scientific rationale.

7. Final Recommendation

In its current form, the protocol addresses a relevant and potentially innovative research question; however, it requires substantial methodological clarification, particularly regarding the sample size calculation and the delimitation of the clinical scope of the proposed findings.

Note: I declare that Google Translate was used exclusively for language translation assistance. No artificial intelligence tools were used in the scientific analysis or evaluation of the manuscript.
