# Supplementary material for: Protocol of a mixed (Retrospective and Prospective) longitudinal observational study of amiodarone for chronic Chagas cardiomyopathy (AMIO-CHAGAS study): Investigating immunomodulatory and trypanocidal effects
Source: PLoS One. 2026 Apr 28;21(4):e0347305. doi: 10.1371/journal.pone.0347305 (PMC13123931; doi:10.1371/journal.pone.0347305)
Supplement: Attachment — Submitted filename: Response to Reviewers.docx [file pone.0347305.s003.docx]

**PLOS ONE Decision:**

**Revision required [PONE-D-25-46528]**

**Journal Requirements:**

**1. Please ensure that your manuscript meets PLOS ONE's style requirements, including those for file naming.**

The manuscript has been revised in accordance with the PLOS ONE guidelines.

**2. Please include your tables as part of your main manuscript and remove the individual files.**

We have included the table as part of the main manuscript and removed it from the individual file.

**3. Your ethics statement should only appear in the Methods section of your manuscript.**

The ethics statement is included only in the Methods section.

**4. Please ensure that you refer to Figure 1 in your text as, if accepted, production will need this reference to link the reader to the figure.**

We apologize for the mistake; Figure 1 has been included in the manuscript text.

**Response to the Editor Comments**

**I consider that the protocol corresponds to experimental design (the effect of AMIO on CCC in Chagas Disease). This involves registering the protocol on a registry of clinical trials. Also, in the protocol is not include a sample of informed consent to verify if the consent includes the destiny of blood samples after the study.**

We appreciate the editor’s comments and the opportunity to clarify these points. We would like to emphasize that the submitted study is an observational clinical study, which includes both retrospective and prospective components. Unlike a clinical trial, our study does not involve the assignment of interventions, randomization of participants, or experimental manipulation of treatment. Instead, it aims to observe and analyze clinical outcomes under routine care conditions.

Because of these fundamental differences, the study does not meet the standard definition of a clinical trial, and therefore registration in a clinical trial registry is not applicable. Observational studies of this nature follow patients and collect data according to standard clinical practice rather than testing the efficacy or safety of an intervention under experimental conditions.

In summary, the study fully complies with ethical standards and methodological requirements for observational research, while not fitting the criteria for a clinical trial registry.

**While revising your submission, please upload your figure files to the Preflight Analysis and Conversion Engine (PACE) digital diagnostic tool,** [**https://pacev2.apexcovantage.com/**](https://track.editorialmanager.com/CL0/https:%2F%2Fpacev2.apexcovantage.com%2F/1/010f0199324ed3fb-729caf16-697f-4d68-b8f8-6ebd91131233-000000/Ow-UXMx4xIr1G7QaDN5SCsbY9ICLpiYMOhwHe5eRTec=228)**.**

As recommended, we uploaded the figures to PACE during the manuscript revision process.
