# Supplementary material for: Protocol of a mixed (Retrospective and Prospective) longitudinal observational study of amiodarone for chronic Chagas cardiomyopathy (AMIO-CHAGAS study): Investigating immunomodulatory and trypanocidal effects
Source: PLoS One. 2026 Apr 28;21(4):e0347305. doi: 10.1371/journal.pone.0347305 (PMC13123931; doi:10.1371/journal.pone.0347305)
Supplement: Attachment — Submitted filename: Response to Reviewers - PONE-D-25-46528R1.docx [file pone.0347305.s004.docx]

**Response to Reviewers - PONE-D-25-46528R1**

**Protocol of a Mixed (Retrospective and Prospective) Longitudinal Observational study of Amiodarone for Chronic Chagas Cardiomyopathy (AMIO-CHAGAS study): Investigating Immunomodulatory and Trypanocidal Effects**

**Reviewer #1:** The AMIO-CHAGAS study protocol is robust, ethically sound, and of high clinical importance. It addresses an underexplored yet highly relevant topic in the management of chronic Chagas cardiomyopathy. The combination of immunological, molecular, and clinical endpoints represents an innovative and comprehensive approach.

**Recommendations for Minor Improvement**

**Clarify exploratory of confirmatory analyses:**

1. **Specify which molecular and immunological measures (e.g., cytokines, gene expression, *T. cruzi* DNA) are confirmatory endpoints linked to the main hypothesis and which are exploratory analyses intended for hypothesis generation. This will improve methodological transparency and interpretability.**

**Response:** We appreciate this suggestion. In the present study, all molecular and immunological measures—including cytokine levels, gene expression, and T. cruzi DNA—are planned as confirmatory endpoints, as they are directly related to the main hypothesis evaluating the immunomodulatory and trypanocidal effects of amiodarone in patients with CCC. These diverse methodologies were intentionally adopted to broaden the range of parameters analyzed, thereby increasing the robustness and interpretability of the results. No analyses are exploratory, and all are designed to address this central research question.

2. **Describe long-term handling of biological samples:**

**Response:** Thank you for this important suggestion. The request has been addressed, and the relevant information has been added to the Methods section under the subsection **“Blood Collection and Isolation of Plasma, Serum and Peripheral Blood Mononuclear Cells (PBMCs)”.** This subsection now describes the procedures for long-term storage, management, and disposal of biological samples.

3. **Indicate how serum, plasma, and PBMCs will be managed after analysis—whether stored in a certified biobank, destroyed, or used for future ethically approved studies. Include brief details on storage duration and data governance.**

**Response:** Thank you for this comment. As previously indicated, the requested information has been included in the Methods section under the subsection **“Blood Collection and Isolation of Plasma, Serum and Peripheral Blood Mononuclear Cells (PBMCs)”.** Biological samples will be stored under institutional responsibility for up to five years after study completion and subsequently discarded according to institutional biosafety regulations. During this period, samples may be used for additional subprojects subject to appropriate ethical approval.

**Refine language and presentation:**

4. **A light English revision is suggested to enhance flow and precision:**

**Response:** Thank you for this suggestion. The manuscript has been carefully revised for English language, grammar, and clarity. All changes have been highlighted in the revised text.

5. **Abstract: simplify and unify tenses.**

**Response:** Thank you for this helpful suggestion. The abstract has been revised to simplify sentence structure.

6. **Discussion: clarify links between preclinical findings and the study rationale.**

**Response:** We thank the reviewer for this valuable suggestion. The Discussion has been revised to more clearly highlight how the clinical observations from the BENEFIT study are supported by experimental evidence from murine models, thereby providing robust support for our hypothesis regarding the potential immunomodulatory and trypanocidal effects of amiodarone in patients with CCC.

7. **Tables/Figures: define all abbreviations in legends.**

**Response:** Thank you for this suggestion. All abbreviations in tables and figure legends have been defined accordingly in the revised manuscript.

Overall, the protocol is scientifically robust, ethically sound, and of high translational relevance. These minor refinements will improve clarity and publication readiness.

**Reviewer #2:** In its current form, the protocol addresses a relevant and potentially innovative research question; however, it requires substantial methodological clarification, particularly regarding the sample size calculation and the delimitation of the clinical scope of the proposed findings

**Scientific Review Report**

The manuscript presents an ambispective longitudinal protocol (retrospective and prospective), entitled AMIO-CHAGAS, which evaluates the use of amiodarone in patients with chronic Chagas cardiomyopathy treated at a referral center in Brazil. Considering that Chagas disease remains endemic in this country and that Chagas cardiomyopathy represents a significant cause of disability and cardiovascular morbidity and mortality, the topic is clinically relevant.

The study population consists of patients with confirmed diagnosis of Chagas disease. The primary objective of the protocol is to evaluate the potential immunomodulatory and trypanocidal effects of amiodarone, exploring whether, in addition to its recognized antiarrhythmic effect, the drug could have an etiological impact on *Trypanosoma cruzi* infection.

**1. Originality and Relevance**

The protocol addresses a pharmacological aspect that has not yet been fully elucidated in humans: the potential immunomodulatory and trypanocidal effects of amiodarone beyond its conventional antiarrhythmic indication. Given that the available clinical evidence regarding this effect remains limited, the study aims to contribute to a relevant gap in current knowledge.

Furthermore, considering the persistent prevalence of Chagas disease and its impact in terms of cardiovascular morbidity and mortality, the exploration of additional effects of drugs already used in this population holds scientific and clinical interest.

**2. Discussion and Clinical Applicability**

Although the protocol proposes an innovative hypothesis regarding the potential etiological effect of amiodarone in humans, it would be advisable for the authors to more thoroughly contextualize the clinical relevance of these findings. In particular, it is important to discuss the drug's safety profile, characterized by known adverse effects, in comparison with currently established etiological therapies for Chagas disease. It is suggested that the clinical scope of the study be more clearly defined, emphasizing the exploratory nature of the research.

**Response:** We thank the reviewer for this valuable suggestion. The manuscript has been updated to address these points, and the relevant information regarding clinical relevance, safety profile, comparison with conventional therapies, and the exploratory nature of the study has been incorporated into the final paragraph of the Discussion.

**3. Methodology**

The methodological design is consistent with the stated objective; however, the proposed sample size (n = 90) may be limited for detecting clinically significant differences, particularly given the exploratory nature of the immunomodulatory and trypanocidal effects being evaluated. Although the authors report having based their estimate on two previous studies and increased the calculated sample size by 20% to account for potential losses, the parameters used for a formal sample size calculation are not described. The absence of this information makes it difficult to determine whether the study will have sufficient statistical power to support robust conclusions.

A more detailed description of the sample size calculation procedure would strengthen the methodological rigor of the protocol.

**Response:** We thank the reviewer for this important comment. We have revised the Methods section to provide additional details regarding the sample size calculation. Specifically, we included the significance level (α), statistical power (1 − β), standard deviations, and the minimum differences considered clinically relevant for the primary and secondary endpoints. The sample size of 90 participants (45 per group) was determined based on the scenario requiring the largest sample, with an additional 20% to account for potential losses, ensuring sufficient statistical power to detect meaningful differences in the inflammatory and echocardiographic parameters evaluated.

**4. Ethical Aspects**

The manuscript states that the study was approved by an institutional ethics committee, including the corresponding approval number. It also specifies that all participants provided informed consent prior to inclusion, and the authors declare the absence of conflicts of interest.

Overall, the protocol appears to comply with fundamental ethical standards for research involving human participants.

**5. Reporting and Data Availability**

The manuscript does not include an explicit statement regarding data availability. It is recommended that a specific section be incorporated clarifying whether the data will be publicly available, accessible upon reasonable request, or subject to ethical restrictions.

**Response:** We thank the reviewer for this important comment. We have updated the “Data Collection” section of the manuscript to clarify that the data generated in this study will be made available upon reasonable request, subject to ethical and privacy considerations. This revision ensures compliance with PLOS ONE’s data sharing policy and provides transparency regarding data accessibility.

**6. Writing and Clarity**

The manuscript is clearly written and allows adequate understanding of the objective and methodological structure of the protocol. The organization is coherent and facilitates comprehension of the scientific rationale.

**7. Final Recommendation**

In its current form, the protocol addresses a relevant and potentially innovative research question; however, it requires substantial methodological clarification, particularly regarding the sample size calculation and the delimitation of the clinical scope of the proposed findings.

Note: I declare that Google Translate was used exclusively for language translation assistance. No artificial intelligence tools were used in the scientific analysis or evaluation of the manuscript.
